# Comparison of thermal traits of *Polistes dominula* and *Polistes gallicus*, two European paper wasps with strongly differing distribution ranges

**DOI:** 10.1007/s00360-016-1041-x

**Published:** 2016-10-15

**Authors:** Helmut Kovac, Helmut Käfer, Iacopo Petrocelli, Anton Stabentheiner

**Affiliations:** 1 Institut für Zoologie, Karl-Franzens-Universität Graz, Universitätsplatz 2, 8010 Graz, Austria; 2 Dipartimento di Biologia, Università degli Studi di Firenze, Via Madonna del Piano 6, 50019 Sesto Fiorentino, Italy

**Keywords:** *Polistes dominula*, *Polistes gallicus*, Thermal limits, Metabolic rate, Body temperature, Environment

## Abstract

The two paper wasps, *Polistes dominula* and *Polistes gallicus*, are related species with strongly differing distribution ranges. We investigated thermal tolerance traits (critical thermal limits and metabolic response to temperature) to gain knowledge about physiological adaptations to their local climate conditions and to get evidence for the reasons of *P. dominula*’s successful dispersion. Body and ambient temperature measurements at the nests revealed behavioural adaptations to microclimate. The species differed clearly in critical thermal minimum (*P. dominula* −1.4 °C, *P. gallicus* −0.4 °C), but not significantly in critical thermal maximum of activity (*P. dominula* 47.1 °C, *P. gallicus* 47.6 °C). The metabolic response did not reveal clear adaptations to climate conditions. At low and high temperatures, the metabolic rate of *P. dominula* was higher, and at intermediate temperatures, we determined higher values in *P. gallicus*. However, the species exhibited remarkably differing thermoregulatory behaviour at the nest. On average, *P. gallicus* tolerated a thoracic temperature up to ~41 °C, whereas *P. dominula* already tried at ~37 °C to keep the thorax below ambient temperature. We suggest this to be an adaptation to the higher mean ambient temperature we measured at the nest during a breeding season. Although we determined for *P. dominula* a 0.5 °C larger thermal tolerance range, we do not presume this parameter to be solely responsible for the successful distribution of *P. dominula*. Additional factors, such as the thermal tolerance of the queens could limit the overwintering success of *P. gallicus* in a harsher climate.

## Introduction

Insect species that have a long history of adaptation to a certain environment are predestined for studying their special physiological adaptations (see e.g. Vorhees et al. 2013). Temperature is a crucial abiotic parameter in an animal’s life, which influences nearly all physiological and biochemical processes. The environmental temperature is determining the distribution patterns of most insect species. Therefore, the insects’ thermal tolerance traits are of special interest concerning their distribution and prediction of future dispersion due to climate warming.

The two paper wasps *P. dominula* and *P. gallicus* are related species from a phylogenetic, biogeographic and ecological point of view, with strongly differing but overlapping distribution ranges. Both originate from the circum-mediterranean area. While *P. gallicus* distribution is mainly limited to its original Mediterranean climate region (Carpenter 1996), *P. dominula* has expanded its distribution range immensely and has settled in regions with harsher climatic conditions (Cervo et al. 2000). *P. dominula* is known to even survive in alpine regions at medium altitudes in Central Europe. Nowadays, it is one of the most abundant *Polistes* species in Europe. Its distribution covers Southern and Central Europe, and possibly due to climate change, it is still expanding its range to the north. It has reached Northern Germany and Denmark in recent times (Pekkarinen 1999; Smit 2003; Woydak 2006), albeit it is absent from the coldest parts of Northern Europe (Cervo et al. 2000). Species with strongly differing distribution ranges provide powerful study systems for understanding adaptation to different environmental conditions. Studying these broadly distributed species will reveal knowledge about adaptations on highly variable environment temperatures. The abundance and success of widely distributed species across variable environments make them suitable models for exploring which traits will be important for resilience to climate change. Furthermore, the findings could show how species will respond to climate change and which traits are indicative of vulnerability (see e.g. Klok and Chown 2003; Terblanche et al. 2006; Hoffmann 2010; Sgrò et al. 2010; Vorhees et al. 2013).

A number of organisms, especially insects, are extending their range in response to the trend of increasingly warmer ambient temperatures. Thermal tolerance may limit and, therefore, predict insects’ geographic distribution. Physiological studies can help predict effects of climate change through delimiting which species currently live closest to their thermal tolerance limits. Thermal tolerance traits include the upper and lower lethal temperatures (see e.g. Bale 1987, 1996; Chown and Nicolson 2004; Sinclair et al. 2006) and a range of non-lethal measures, the critical thermal limits (see e.g. Gaston and Chown 1999; Hazell and Bale 2011; MacMillan and Sinclair 2011; Gallego et al. 2016). The upper and lower lethal temperatures constrain the thermal range of respiration and activity. Of the non-lethal thermal tolerance measures, the index most commonly employed by researchers is the critical thermal temperature or coma temperature (critical thermal limits: CT_min_ and CT_max_, chill coma and heat coma, respectively). When temperatures increase above the optimal range, performance declines rapidly to the critical thermal maximum (CT_max_), the high temperature at which the animals cease to function. When temperatures decrease below the optimal range, performance declines more slowly to the critical thermal minimum (CT_min_) or chill coma. The temperature range between CT_max_ and CT_min_ is often referred to as thermal tolerance range (Huey and Stevenson 1979).

The “thermolimit respirometry” is a standardized method for determining the upper thermal limit of normal respiratory function (respiratory CT_max_, Lighton and Turner 2004). A combination of this technique with conventional behavioural assessment of the critical thermal maximum (activity CT_max_), e.g., by using detection of movement by means of video analysis or infrared diode actigraphy, objectively pinpoints the exact temperature of short-term physiological failure (see e.g. Klok et al. 2004; Lighton and Turner 2004; Stevens et al. 2010; Käfer et al. 2012).

The standard metabolic rate (SMR), usually equated with resting metabolism, is a very important parameter in insects’ life representing the energetic costs of simple subsistence, determining an individual’s minimum energy requirement under a standardized set of conditions. It is fundamental for comparing the relative energy expenditures of particular activities. The SMR enables the comparison and assessment of basic energetic costs across species. Ideally, measurements of SMR are made under constant environmental conditions on individuals of known mass, sex and age that show no external activity (Hack 1997). The resting metabolism of *P. dominula* has been thoroughly investigated recently by Käfer et al. (2015). A dependence of the CO_2_ release on ambient temperature in an exponential shape has been described. The standard metabolic rate is presumed to reflect the energetic costs of adaptation to a particular thermal environment (see e.g. Clarke 2004; Clarke and Fraser 2004; Watson et al. 2014; Magozzi and Calosi 2014; Tomlinson and Menz 2015; Tomlinson and Phillips 2015) and is an ideal parameter to compare the two paper wasps concerning their basic energetic expenditure.

The specific aim of this study was to investigate thermal tolerance traits of two related species, to gain knowledge about physiological adaptations to their environmental conditions. In a first step, the thermal traits of workers were investigated, as they are responsible for the successful development of the colony during the breeding season and a sufficient rearing of reproductive queens and males. Results should give evidence for the reason of *P. dominula’*s successful distribution in cooler climate areas and enable prognosis about future dispersion. Therefore, the critical thermal limits and the metabolic response in a range of temperatures were measured. In many insect studies, it has often been found that variability in thermal tolerance within and among species correlates to the environmental temperatures experienced by the populations or species (see e.g. David et al. 2003; Ayrinhac et al. 2004; Terblanche et al. 2008; Overgaard et al. 2011; Hoffmann et al. 2012). To determine the actual microclimate conditions, the two *Polistes* species are exposed to during a breeding season, we measured the ambient air temperature at the nest. Additional measurements of the wasps’ body temperature should show their actual temperature at a certain (high) ambient temperature and reveal special thermoregulatory behaviour. The results of the critical thermal maximum tests will enable predictions whether they will be able to deal with high temperature extremes due to ongoing climate change. Metabolic data could reveal adaptation to climate conditions and enable predictions about whether their energy economy changes in a changing environment. In a changing environment due to global climate warming, the knowledge of such basal physiological parameters is essential to predict the animals’ capability to cope with future-predicted environmental conditions.

## Materials and methods

### Animals, study sites and environmental conditions

To show the climatic variability of the two climate regions, data of the meteorological stations (period 1971–2000) of Graz (N47° 04′ 40″ E15° 26′ 56″) and Florence (Firenze) (N43° 46′ 28″ E11° 15′ 18″) were evaluated (Origin of data for Graz: Zentralanstalt für Meteorologie und Geodynamik, http://www.zamg.ac.at/fix/klima/oe71-00/klima2000/klimadaten_oesterreich_1971_frame1.htm, and for Florence (Firenze): LaMMA Consortium, http://www.lamma.rete.toscana.it/clima-e-energia/climatologia/clima-firenze-1971-2000).

The mean annual temperature in Graz was 9.4 °C and in Florence 14.9 °C. They differed also in the mean maximum and minimum temperatures (Graz: mt_max_ = 14.6 and mt_min_ = 5.5 °C, Florence: mt_max_ = 20.5 and mt_min_ = 9.3 °C).

The environmental conditions in the habitat were determined during the 2013 breeding season at two different nests per each paper wasp species (*Polistes dominula, P. gallicus*). The nests of *P. dominula* were attached on roofing tiles inside a loft of an old house in Gschwendt (Styria, Austria), whereas the nests of *P. gallicus* were located in the recess of a window (outside, in shade) at the Department of Biology of the University of Florence in Sesto Fiorentino (Tuscany, Italy). The ambient air temperature was measured about 1 cm above the nests with NiCr/Ni thermocouples and outside of the loft in shade. The temperature data were logged at an interval of 15 min with ALMEMO 2290-8 data loggers (Ahlborn GmbH, Holzkirchen, Germany) or Extech Temperatur Datenlogger SD 200 (FLIR Commercial Systems, Nashua, New Hampshire, USA). For a further analysis of the temperature recordings, the data were divided in 1 °C intervals and the frequency of temperature classes was counted. By means of the Sinclair microclimate macro V 2.0.1 (Sinclair 2001), the number of consecutive intervals (interval duration 15 min) was evaluated, when a special threshold (40–47 °C) was exceeded.

For the experiments on thermal limits and metabolic rate, adult female workers were collected in summer 2013–2015. *P. gallicus* were collected at the nests in Sesto Fiorentino, Italy. *P. dominula* were also collected at the nests or from flowers in an orchard in Gschwendt, Austria. The wasps were weighed before the experiments with a balance to the nearest of 0.1 mg (AB104, METTLER-TOLEDO, Greifensee, Switzerland).

### Critical thermal minimum (CT_min_)

Twenty wasps of each species (*P. dominula*, *P. gallicus*) were captured from at least four different nests. Single wasps were inserted into plastic vials with a volume of 15.3 ml (14 mm diameter × 100 mm length). One (empty) vial was equipped with a thermocouple connected to a data logger (ALMEMO 2890-9, Ahlborn GmbH, Holzkirchen, Germany) which recorded the temperature the animals were exposed to during the experiment. The vials were fixed in a self-constructed shaking device and submerged into a water bath (Julabo F33, JULABO Labortechnik GmbH, Seelbach, Germany). After 5 min of habituation we drove a temperature ramp from 15 °C to –5 °C with a dT of 0.25 °C min^−1^. The minimum temperature was maintained for 5 min, and then the temperature was increased again to 15 °C within 10 min (dT of ~2 °C min^−1^). The individuals inside the vials were forcefully shaken for one second in 1-min intervals during the entire experiment (about 100 min). The experiments were recorded on video for later evaluation (Sony HDR-CX730E, Sony Europe Limited, Wien, Austria). The last-appearance time of movements of antennae or legs after the shaking (according to Andersen et al. 2015) was determined via behavioural observation, and the temperature at that time (critical thermal minimum, CT_min_) was extracted from the logger file.

The thermal tolerance range for each species was calculated as the difference between CT_max_ and CT_min_ (according to Terblanche et al. 2007).

### Critical thermal maximum (CT_max_)

As the combination of respirometry data and activity detection had shown the most accurate results in previous studies concerning the upper thermal maximum (Klok et al. 2004; Lighton and Turner 2004; Stevens et al. 2010; Käfer et al. 2012); respiration and activity as well as body surface temperatures were assessed simultaneously via flow-through respirometry and infrared thermography (Stabentheiner et al. 2012). The critical thermal maximum (CT_max_) of 20 adult females of each species was assessed following a standardized method of driving a temperature ramp from 25° to 55 °C at a dT = 0.25 °C min^−1^ (see e.g. Chown et al. 2009; Stevens et al. 2010; Terblanche et al. 2007). The CT_max_ was defined via observation of activity (activity CT_max_). The point of time when controlled motoric activity ceased and muscle spasms started was determined via behavioural observation, and the temperature at that time was extracted from the logger file (for further information see also Hazell et al. 2008; Klok and Chown 1997; Lighton and Turner 2004; Lutterschmidt and Hutchison 1997). Also, the CT_max_ was determined by thermolimit respirometry (respiratory CT_max_, point of time when cyclic gas exchange ceased, according to Lighton and Turner 2004). The absolute difference sum of CO_2_ production (rADS) is a measure of cumulative dynamic variability (Lighton and Turner 2004). To determine the respiratory CT_max_ more accurately, the inflection point of the rADS residual values from 10 min before to 10 min after the suggested activity CT_max_ was determined. This inflection point helps to determine the minute point of the respiratory CT_max_. The wasps were filmed during experiments with an infrared thermography camera, and the wasps’ body temperature was determined afterwards (for further details of infrared measurement see below). We used thermal ramping to ensure that our data provide ecologically relevant measures of thermal tolerance (see Terblanche et al. 2011) and remain comparable to a broad set of insect studies. For detailed information on the procedure, see Stabentheiner et al. (2012), Käfer et al. (2012) and chapter 6 in Hartfelder et al. (2013).

### Body temperature

Measurements were conducted at five nests of each species, of *P. gallicus* in Sesto Fiorentino (near Florence), Italy, and of *P. dominula* in Gschwendt (near Graz), Austria, respectively. The surface temperature of the wasps (head, thorax, abdomen) was measured by infrared technology (i60, T650sc, FLIR Systems Inc., Danderyd, Sweden). The measured body temperature was calibrated to ~0.7 °C accuracy, assuming a wasp cuticle infrared emissivity of 0.97 (Kovac and Stabentheiner 1999) and using a proprietary Peltier-driven reference source of known temperature and emissivity for camera calibration (for details see Schmaranzer and Stabentheiner 1988; Stabentheiner and Schmaranzer 1987; Stabentheiner et al. 2012). Infrared data were stored digitally on an internal memory card and evaluated afterwards in the laboratory. Evaluation of the surface temperatures of head (*T*_hd_), thorax (*T*_th_), and abdomen (*T*_ab_) was done with AGEMA Research software (FLIR Systems Inc., Wilsonville, USA) controlled by a proprietary Excel (Microsoft Corporation, Redmond, USA) VBA macro. The actual ambient temperature beside the wasps (within ~1–2 cm) was measured with thermocouples connected to a data logger (ALMEMO 2690, Ahlborn GmbH, Holzkirchen, Germany).

### Respiration measurement—resting and active metabolic rate

To determine the wasps’ resting metabolic rate (RMR or standard metabolic rate), we measured the carbon dioxide emission using flow-through respirometry as previously described by Käfer et al. (2012, 2015). The experiments described here were conducted according to the same procedure with the same experimental setup (see also Stabentheiner et al. 2012). Briefly, individual wasps were placed into a respirometry measurement chamber where they were allowed to move freely. Since experiments lasted overnight, they were provided with 1 M sucrose solution. The brass chamber (outer dimension: 6 × 10 × 4 cm, inner dimension: 3 × 3 × 2 cm, volume ~18 ml) was immersed in a water bath (Julabo F33, JULABO Labortechnik GmbH, Seelbach, Germany) for temperature control (accuracy 0.1 °C). The relative humidity (rH) was maintained at 50 % (see Stabentheiner et al. 2012 for details). The experimental ambient temperature (*T*_a_) for the wasps was set to 15, 25 or 35 °C in these experiments (for other temperatures see below). However, the actual ambient air temperature could deviate slightly from these settings, because the measurement chamber was not completely submerged to allow observation with an infrared thermography camera (see below). The exact temperature was, therefore, measured in the respirometry chamber near the wasps (within ~1–2 cm) with a NiCr/Ni thermocouple. Temperature data were recorded at one-second intervals with an ALMEMO 2890-9 data logger (Ahlborn GmbH, Holzkirchen, Germany). Each wasp was tested at one temperature and was used for one experiment only. In each temperature category, 5–12 wasps were investigated, and the data evaluation was done according to the temperature categories.

The insects’ CO_2_ release was measured with a differential infrared carbon dioxide gas analyser (DIRGA, URAS 14, ABB), with an accuracy of ~2 ppm. To maximize the system sensitivity (<0.2 ppm), the air was taken from outside the laboratory. Before it entered the reference tube of the DIRGA, the air was pumped through a 10l container to dampen fluctuations in CO_2_ content, passed the pump and mass flow controllers (0–1000 ml min^−1^, Brooks 5850 S), and then passed another container (5 l) for additional CO_2_ and pressure fluctuation damping. The air was dried by passing it through two Peltier-driven cool traps (10 °C) before it entered the URAS reference and measurement tubes (where it was heated to 60 °C). The airflow in the system was 144 ml min^−1^. The volumes (nl) of CO_2_ production reported in this paper refer to standard (STPS) conditions (0 °C, 101.32 kPa = 760 Torr). The CO_2_ release was recorded at one-second intervals. At the beginning and at the end of each experimental run and at an interval of 3 h during experiments, the gas analyser was calibrated automatically in zero and end point by the use of internal calibration cuvettes, and the data were corrected for any remaining drift or offset (for further methodical details, see Stabentheiner et al. 2012).

To control the wasps during these experiments, the brass measurement chamber was covered by a transparent plastic film which allowed observing the wasps and recording their behaviour and temperature with an infrared thermography camera (ThermaCam SC2000 NTS, FLIR). The plastic film was transparent in the infrared range from 3 to 13 µm and allowed thermographic measurements of the wasp’ body surface temperature. Endothermy may increase the energy turnover considerably above the resting level. Therefore, the thoracic temperature excess (*T*_excess_ = *T*_thorax_ − *T*_abdomen_) was used as a measure to assess the wasps’ degree of endothermy. The infrared video sequences allowed quantification of an even small endothermic state of wasps over a longer resting period. In addition, infrared thermography allowed detection of cooling efforts. The activity and behaviour of the wasps was analysed afterwards from the infrared video sequences.

The RMR data of *P. dominula* were extracted from the paper of Käfer et al. (2015), with the exception of the data at 5, 40 and 45 °C, which were made 2013–2015 during the experimental series for *P. gallicus*. The measurements at these experimental temperatures were conducted with the same experimental setup as mentioned above, extended with an eight-channel multiplexer (RM Gas Flow Multiplexer, Sable Systems, Las Vegas, Nevada, USA), using stop-flow respirometry. The multiplexer controlled the sequential flushing and closing of the metabolic chambers (plastic vials 10 × 30 mm inner dimension, ~2.4 ml volume), which allowed the simultaneous measurement of eight individuals. During the flush phase, the metabolic chambers were perfused with humidified air (50 % rH) at a fixed rate of 144 ml min^−1^. After the flush phase, the metabolic chamber was closed. The remaining eight chambers were flushed sequentially in a similar manner. The duration of the flushing phase of one chamber was 1 min; therefore, the closed phase was 7 min. Experiments lasted for about 60 min (40 °C) or 30 min (45 °C) till the wasps showed first spasms.

Our definition of “resting” was: no or only small visible signs of activity, i.e. only movements of antennae or single legs allowed (according to Crailsheim et al. 1999; Stabentheiner and Crailsheim 1999; Stabentheiner et al. 2003; Kovac et al. 2007; Käfer et al. 2012). However, the data at 40 and 45 °C represent values of an active metabolism, since the wasps never were at rest at these high temperatures. The experiments conducted at 5, 40 and 45 °C allowed no body temperature measurement by infrared thermography, as the test tubes were submerged in the water bath.

### Data analysis and statistics

For further data analysis, the evaluated resting phases were divided into 10-min intervals. In some individuals, at high *T*_a_, the duration of resting phases decreased to such an extent that we had to reduce the interval to a minimum of 5 min. For the evaluated intervals, the mean CO_2_ production rate (VCO_2_) was calculated. This evaluation procedure concerns measurements at 15, 25 and 35 °C. During measurements at 5 °C, the wasps remained calm during the entire experiment. At 40 and 45 °C, the wasps were always agitated and never calmed down, so we could determine their active metabolic rate only at these high ambient temperatures. To distinguish between periods of activity and rest, behavioural observation was conducted via simultaneous video recordings (Sony HDR-CX730E, Sony Europe Limited, Wien, Austria) of all eight measurement chambers.

Data analysis and statistics were done in Excel (Microsoft Corporation, Redmond, USA) with custom-made peak-finding formulas and VBA macros, and with Origin software (Origin 8.1, OriginLab Corporation, Northampton, USA) and Statgraphics Centurion XVI (StatPoint Technologies Inc., Warrenton, USA). Regression lines were compared and tested using ANOVA.

For comparison of the two species, *P. gallicus* and *P. dominula*, a subset of the RMR data (*T*_a_ 15, 25, 35 °C, according to our temperature categories) from the paper of Käfer et al. (2015) was extracted and presented in Table 2 and Fig. 4. One experiment (from Käfer et al. 2015 *P. dominula* at 25 °C, Fig. 4b) lacks the body temperature measurement due to a technical failure.

## Results

### Critical thermal minimum (CT_min_)

The wasps calmed down very quickly after insertion of the vials into the water bath with the starting temperature of 15 °C. After starting the experiment and stimulation by shaking, they exhibited movements of legs and antennae. The temperature when these movements ceased was determined as the critical thermal minimum (CT_min_), which is the onset of chill coma. It differed significantly between the two species. We determined for *P. dominula* a mean value of −1.4 ± 1.29 °C, and for *P. gallicus* −0.4 ± 0.65 °C (*n* = 20 each; *P* < 0.01, *t* test, Table 1).

**Table 1:** Statistical details of critical thermal maxima and minima of activity (act. CT_max_, act. CT_min_) and respiratory critical thermal maxima (resp. CT_max_) of *P. dominula* and *P. gallicus*

	Act. CT_max_	SD	Max	Min	*N*	Resp. CT_max_	SD	Max	Min	*N*
*P. dominula*	47.1	1.2	49.6	44.3	19	47.4	1.1	49.6	45.6	20
*P. gallicus*	47.6	1.2	49.7	45.7	17	47.7	1.0	49.4	46.1	20
P (*t* test)	0.24427					0.40115				

	Act. CT_min_	SD	Max	Min	*N*	Resp. CT_min_	SD	Max	Min	*N*
*P. dominula*	−1.4	1.3	0.4	−4.3	20					
*P. gallicus*	0.4	0.7	0.8	−1.8	20					
*P* (*t* test)	0.01									

Means were compared with t test

*SD* standard deviation, *N* number of experiments (wasps)

### Critical thermal maximum (CT_max_)

**Fig. 1:**
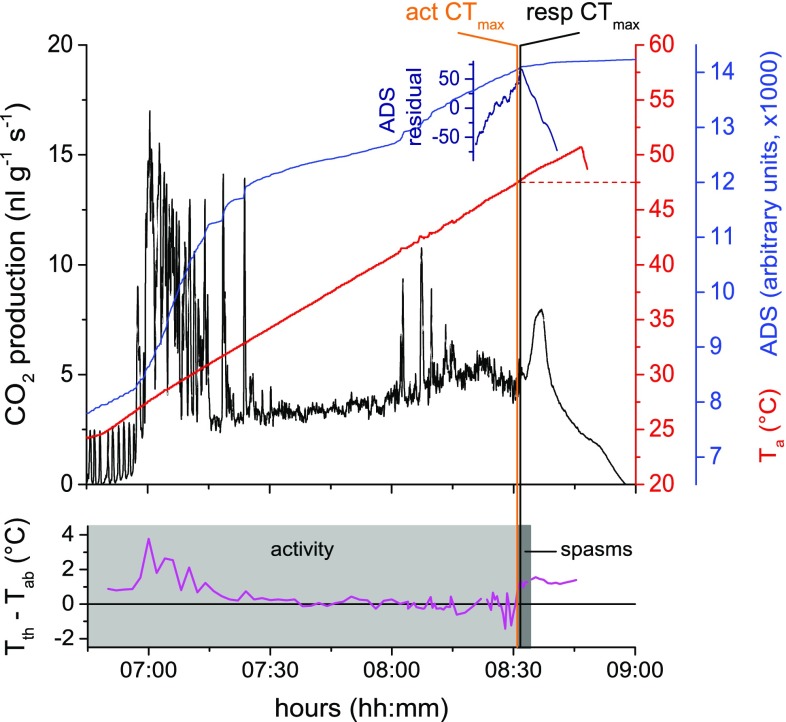
CO_2_ production, activity and thoracic temperature excess of *P. gallicus* during a thermolimit experiment. Respiratory CT_max_ = 47.7 °C, activity CT_max_ = 47.5 °C. Activity CT_max_ was indicated by cease of controlled motoric activity (=mortal fall). Residual analysis of the absolute difference sum (rADS) of CO_2_ indicated the point of respiratory CT_max_ (=cease of cyclic respiration, see “Materials and methods”)

Twenty trials for determining the upper critical thermal maximum (CT_max_) of activity and respiration were performed for each species. Figure 1 shows a representative thermolimit experiment of an individual of *P. gallicus*. The wasps were rather calm at the moderate temperature range and became active when the ambient air temperature exceeded 30 °C. If the temperature reached about 40 °C, they became very excited and tried to escape from the measurement chamber. The lower part of Fig. 1 shows the thermal reaction (thorax temperature excess: *T*_excess_ = *T*_thorax_ − *T*_abdomen_) of the same wasp, with an excess temperature reaching nearly 4 °C when the wasp was agitated. At very high temperatures of about 45 °C, they often performed cooling behaviour with regurgitated fluid droplets. Coordinated body movements ceased with the mortal fall when the wasps died. The averaged values of the mortal fall provided the knockdown temperature (see Klok et al. 2004; Stevens et al. 2010; Käfer et al. 2012) or activity CT_max_ (for *P. dominula*: 47.1 °C, *P. gallicus*: 47.6 °C, respectively; see Table 1). After the initial high activity and, therefore, metabolic rate, the CO_2_ trace showed a typical progression, followed by a distinct post-mortal plateau after the respiratory CT_max_. The respiratory CT_max_ was determined via the inflection point of the rADS residual values 10 min before and after the mortal fall. Cyclic respiration finished in *P. dominula* at 47.4 °C and in *P. gallicus* at 47.7 °C. The CT_max_ of activity as well as respiration was somewhat higher in *P. gallicus*, but did not differ significantly from *P. dominula* (*P* > 0.05, *t* test; see Table 1). There was no significant difference (*P* > 0.05, *t* test; see Table 1) between the two methods, activity and respiration, for determining the critical thermal maximum.

The thermal tolerance range for each species calculated as the difference between CT_max_ and CT_min_ was 48.5 °C in *P. dominula* and 48.0 °C in *P. gallicus*.

### Body temperature at the nest

**Fig. 2:**
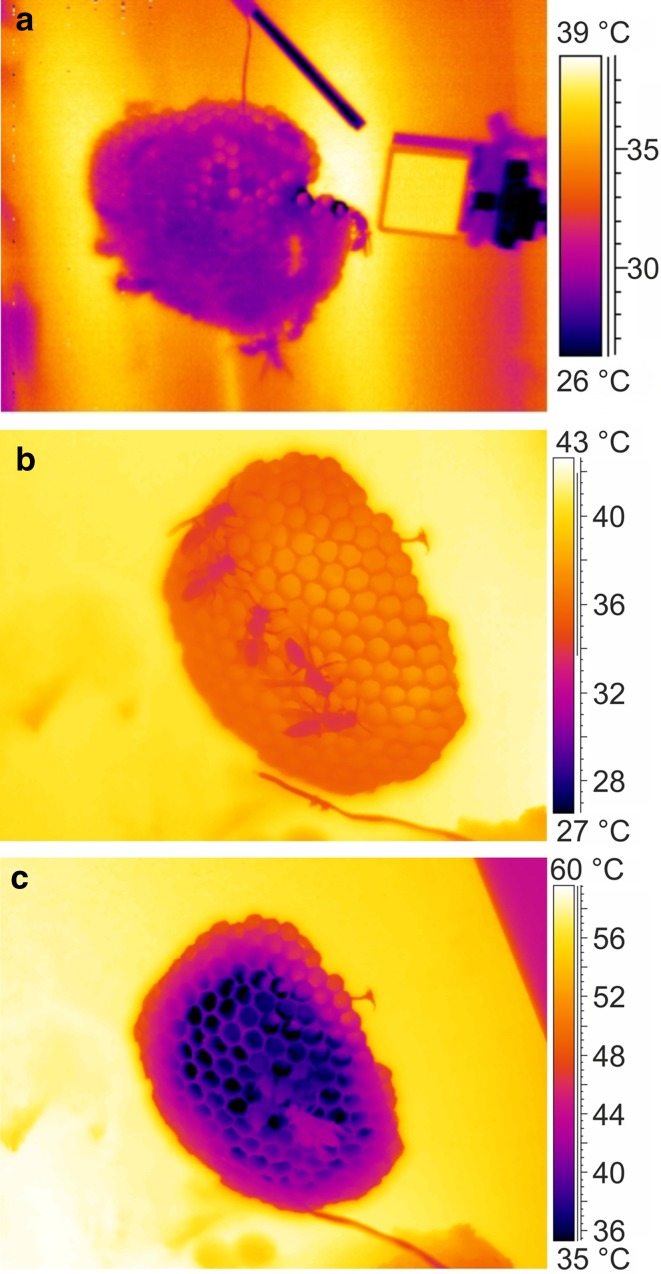
Thermograms of a nest of *P. dominula* at the location in (**a)** Austria at *T*_a_ ~ 32 °C, and of *P. gallicus* at the location in Italy at (**b)** *T*_a_ ~ 33 °C and at (**c)** *T*_a_ ~ 37 °C; note actively cooled nest centre (*dark area*) with four wasps cooling their heads inside the cells, a regularly observed behaviour at these high temperatures

**Fig. 3:**
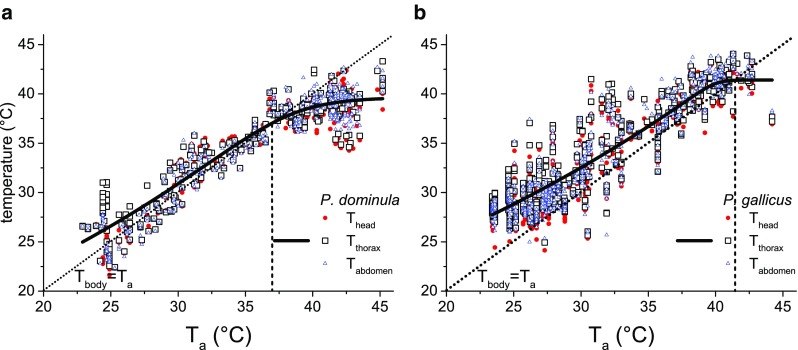
Surface temperature of head, thorax and abdomen of individuals of *P. dominula* (**a**) and *P. gallicus* (**b**) measured at the nest in dependence on ambient temperature. The *vertical line* represents the value when the interpolated thorax temperature equals the ambient temperature. The thorax temperature was fitted with a sigmoidal function, *T*_thorax_ = a*[1 + (d − 1)*exp^−k*(Ta − xc)^]^1/(1 − d)^. Parameters for *P. dominula*: *a* = 39.62378, xc = 32.21605, *d* = 15.95662, *k* = 0.45019 (adj. *R* ^2^ = 0.89353, *N* = 339, 5 nests); *P. gallicus*: *a* = 41.40934, xc = 37.66565, *d* = 78.32908, *k* = 1.86841 (adj. *R* ^2^ = 0.83244, *N* = 675, 5 nests)

The wasps’ body surface temperature was mostly near the ambient air temperature (*T*_a_) measured at the nest (Fig. 2 and 3) and increased with *T*_a_. The thoracic temperature was best described with a sigmoidal fit function (*T*_thorax_ = a*[1 + (*d* − 1)*exp^−k*(Ta − xc)^]^1/(1 − d)^). At lower temperature, the thorax temperature was above the ambient air, and the difference between ambient and thorax temperature was greater than at higher *T*_a_ (*T*_a_ = 25 °C, *T*_thorax_ − *T*_a_: *P. gallicus* 3.8 °C, *P. dominula* 1.6 °C, respectively). At higher temperatures, in *P. gallicus* the mean thorax temperature approximated to the ambient air at 41.4 °C (evaluated from the sigmoidal function), whereas in *P. dominula* this point was reached already at 37.0 °C; their mean thorax temperature at 41.4 °C already was 2.4 °C below *T*_a_. Linear regression lines calculated for the species’ thorax temperature differed significantly from each other (model *P* < 0.0001, F-quotient = 1889.92; *T*_a_: F-quotient = 5443.55, *P* < 0.0001; intercept: F-quotient = 218.69, *P* < 0.0001; slope: F-quotient = 7.51, *P* = 0.0061; ANOVA). The temperature of head and abdomen were quite similar to that of the thorax or slightly below (Fig. 3). Active cooling of the nest with water droplets was observed in both species at high *T*_a_ (>~30 °C). Figure 2c shows an example in *P. gallicus*. Though fanning behaviour is known as a measure against (nest) overheating in *Polistes* species (e.g. Steiner 1930; Höcherl et al. 2016), we could not observe this behaviour in *P. gallicus* even at the highest ambient temperatures (>37 °C). To cool their own body, these wasps regularly inserted their heads and thorax into cells already cooled by evaporation of water (Fig. 2c), remaining there motionless until ambient temperature decreased again.

### Metabolism

*Weight and activity* The two species differed significantly in weight from each other. *P. dominula* used for experiments weighed on average 0.080 ± 0.015 g (*N* = 44) and *P. gallicus* on average 0.044 ± 0.011 g (*N* = 48; *P* < 0.0001, *t* test).

Infrared and conventional video sequences enabled us to evaluate the behaviour of the wasps. At temperatures of 15 °C and below, the individuals soon calmed down after insertion into the measurement chamber and remained at rest for most of the experiment. However, above 15 °C—although deprived of light as external stimulus—they were not always motionless. Individuals sometimes moved, walked or fed. Some wasps were not inactive for ten consecutive minutes, especially at high ambient temperatures (~35 °C). At an experimental temperature of 40 and 45 °C, the wasps were very active and permanently tried to escape the measurement chamber (therefore “active metabolic rate”). At 45 °C, many of them died about 30 min after start of the experiments.

**Fig. 4:**
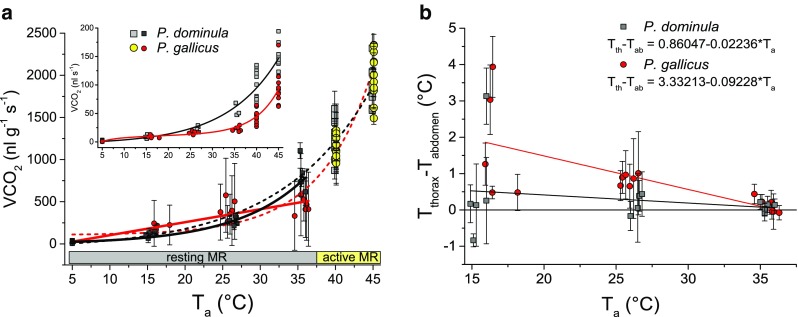
**a** Resting and active metabolic rate (resting MR, *T*_a_ = 5–35 °C; active MR, *T*_a_ = 40–45 °C) of *P. dominula* and *P. gallicus* in dependence on ambient temperature. Data represent means and standard deviation of individuals standardized by allometric scaling of individual body mass (mean ± SD). *Fit curves* of the resting MR and the resting MR + the active MR are shown. RMR of *P. dominula*: VCO_2_ = −3.10356 + 15.36177*exp^(0.10942*Ta)^. RMR of *P. gallicus*: VCO_2_ = –50.69017 + 15.39015*T_a_. RMR + active MR of *P. dominula*: VCO_2_ = −65.626 + 46.443*exp^(0.08296*Ta)^. RMR + active MR of *P. gallicus*: VCO_2_ = 104.58839 + 3.19731*exp^(0.14276*Ta)^. Insert: Resting and active metabolic rate of *P. dominula* and *P. gallicus* in dependence on ambient temperature. Data represent means of individuals. Fit curves for *P. dominula*: VCO_2_ = −4.22912 + 3.29715*exp^(0.08501*Ta)^ *P. gallicus*: VCO_2_ = 21.44251 + 0.02328*exp^(0.18085*Ta)^ − exp(1)/(0.0827*ln(*T*_a_ − 0.2251)). **b** Thorax temperature excess (*T*_thorax_ − *T*_abdomen_) of individuals of *P. dominula* and *P. gallicus* during metabolic measurements in dependence on ambient temperature (*T*_a_ = 15–35 °C). Data represent means and standard deviation (mean ± SD)

*Body temperature* The body temperature of *P. gallicus* individuals during the resting phases (*T*_a_ 15, 25, 35 °C) was evaluated from infrared video sequences and compared with data from *P. dominula*, investigated with the same experimental setup in the same procedure and published recently by Käfer et al. (2015). The wasps’ thoracic temperature excess (T_excess_ = T_thorax_ − T_abdomen_) was highest at 15 °C, and also rather variable, with the greatest standard deviations at this temperature (Fig. 4b, Table 2). This indicates that the wasps sometimes switched between an ecto- and endothermic state. The mean temperature excess at 15 °C was 1.8 °C in *P. gallicus* and 0.6 °C in *P. dominula*, but the difference was not significant (*P*> 0.05, *t* test). At 25 °C, the temperature excess was considerably lower and differed significantly between the two species (*P. dominula*: 0.2 °C, *P. gallicus* 0.8 °C; *P* < 0.01, *t* test). At 35 °C, the temperature excess had reduced to 0.1 °C in both species (*P* > 0.05, t test). Analysis with an ANOVA revealed that the thoracic temperature excess regression lines differ significantly (model *P* = 0.0046, F-quotient = 5.46; *T*_a_: F-quotient = 8.53, *P* = 0.007; intercept: F-quotient = 4.46, *P* = 0.044; slope: F-quotient = 3.37, *P* = 0.0773; Fig. 4b). Individuals of *P. dominula* were less often endothermic during resting phases than *P. gallicus*.

**Table 2:** Mean values and standard deviation of thorax temperature excess (T_thorax_ - T_abdomen_) and metabolic rate (VCO_2_) of *P. dominula* and *P. gallicus* at five temperature categories

	*T*_th_ − *T*_ab_ (°C)					
	5 °C	15 °C	25 °C	35 °C	40 °C	45 °C
*P. dominula*		0.6 ± 1.50 (5)	0.2 ± 0.29 (4)	0.1 ± 0.13 (5)		
*P. gallicus*		1.8 ± 1.57 (5)	**0.8** ± **0.15** (6)***	0.1 ± 0.21 (6)		

	VCO_2_ (nl g^−1^ s^−1^)					
	5 °C	15 °C	25 °C	35 °C	40 °C	45 °C
*P. dominula*	**22.0** ± **11.26** (8)	112.3 ± 31.24 (5)	275.9 ± 48.38 (5)	**808.0** ± **184.52** (5)	1257.6 ± 216.39 (11)	1857.2 ± 222.82 (10)
*P. gallicus*	11.3 ± 2.19 (8)*	**199.2** ± **41.81** (5)**	**411.3** ± **112.56** (6)*	455.0 ± 90.44 (6) **	1115.9 ± 138.21 (11)	2073.7 ± 374.77 (12)

Significant differences are in bold lettering and indicated by * *P* < 0.05, ** *P* < 0.01, *** *P* < 0.001

*N* number of wasps

*Metabolic rate:* The insert in Fig. 4a shows the individuals’ absolute metabolic rate. The two species differed considerably, especially at the lowest (5 °C) and at high (>20 °C) ambient temperature. Due to the significant difference in the wasps’ weight (see above), we decided to use the mass-specific resting metabolic rate (RMR) for further comparison. For comparison of the resting metabolic rate of *P. dominula* with *P. gallicus*, a subset (*T*_a_ 15, 25, 35 °C) of the data from Käfer et al. (2015) was extracted which is presented in Table 2 and Fig. 4a. The wasps’ RMR increased with ambient temperature, following a linear progression in *P. gallicus*. However, in *P. dominula*, RMR increased exponentially with *T*_a_. ANOVA revealed that *P. gallicus* differed significantly from *P. dominula* in RMR (*T*_a_ 5–35 °C; model *P* < 0.0001, F-quotient = 75.67; *T*_a_: F-quotient = 169.28, *P* < 0.0001; intercept: F-quotient = 1.08, *P* = 0.3046; slope: F-quotient = 6.65, *P* = 0.0133; *df* = 2). As the values of *P. dominula* did not increase linearly with *T*_a_, we additionally compared the mean RMR values of each temperature category and obtained always significant differences between the two species (*P* < 0.05, *t* test; Table 2) but not in a consistent way. Values at 5 °C were 48.6 % lower in *P. gallicus.* At 15 and 25 °C, experiments yielded 77.4 and 49.1 % higher values for *P. gallicus*, and at 35 °C, *P. gallicus* had a metabolic rate 43.7 % lower.

At experimental temperatures of 40 and 45 °C, the wasps never calmed down; therefore, these values represent their active metabolic rate. The mean CO_2_ release at 40 °C was, in *P. dominula* 1257.6 ± 216.39 nl g^−1^ s^−1^ and in *P. gallicus* 1115.9 ± 138.21 nl g^−1^ s^−1^, and at 45 °C 1857.2 ± 222.82 and 2073.7 ± 374.77 nl g^−1^ s^−1^, respectively. The active metabolic rate did not differ significantly between the two species (*P* > 0.05, t test; Table 2; Fig. 4a). Across the whole investigated temperature range (*T*_a_ 5–45 °C), the metabolism increased in both species in a similar exponential way (Fig. 4a). ANOVA revealed no clear difference between species (*T*_a_ 5–45 °C, model: *P* < 0.0001, F-quotient = 129.54; *T*_a_: F-quotient = 256.42, *P* < 0.0001; intercept: F-quotient = 0.00, *P* = 0.9738; slope: F-quotient = 0.08, *P* = 0.7722; *df* = 2).

### Climate conditions and microclimate at the nest

**Fig. 5:**
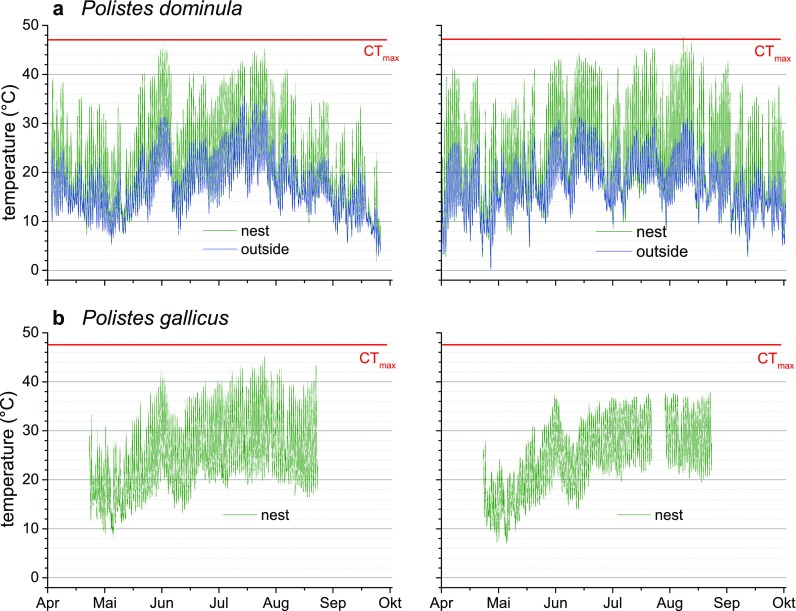
Ambient temperature recordings at two nests of *P. dominula* (**a**) in Gschwendt/Austria and *P. gallicus* (**b**) in Sesto Fiorentino/Italy during a breeding season in 2013. The nests of *P. dominula* were located in a loft and the nests of *P. gallicus* were attached at a recess of a window

The general climate data of the two regions, Mediterranean and temperate climate, differed considerably. The mean annual temperature (period 1971–2000) was 9.4 °C in Graz and 14.6 °C in Florence. The breeding season of *P. dominula* at the location in Austria was similar as observed by Höcherl and Tautz (2015) in Germany. The microclimate measurements during a breeding season at representative nests of *P. dominula* and *P. gallicus* are shown in Fig. 5. The highest ambient temperature measured at the *P. dominula* nest was 47.6 °C. This maximum temperature was 0.5 °C higher than the wasps’ activity CT_max_ (Table 1). The temperature outside the loft (in shade) where the *P. dominula* nests were located was most of the time lower than at the nest inside the loft. Maximum temperature measured at the *P. gallicus* nests was 45.3 °C, which is 2.3 °C lower than *P. gallicus*’ activity CT_max_. This also resembles outside temperature, because these nests were built in the open.

**Fig. 6:**
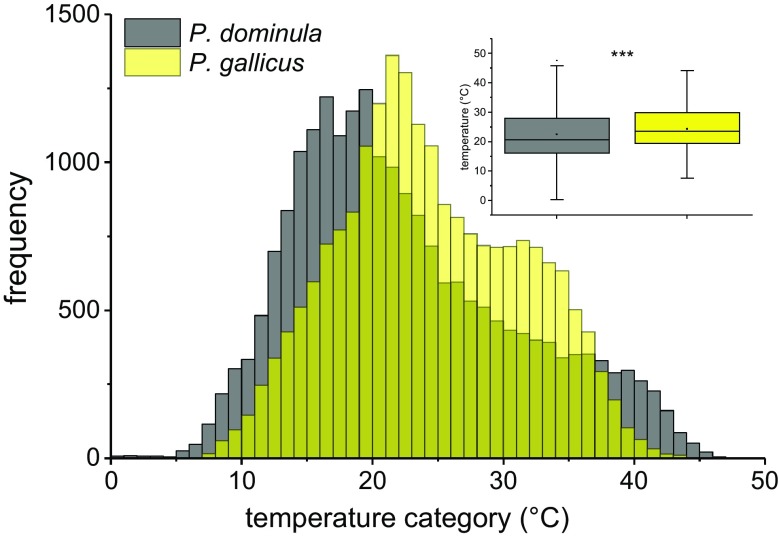
Frequency of temperature intervals (1 °C) measured at two nests of *P. dominula* and *P. gallicus* during a breeding season in 2013 (14 May to 03 September). *Insert*: *Box* and *whisker plots* represent median temperatures with first and third quartiles, and maximum and minimum values measured at the nests, significant difference is indicated by ***P < 0.001 (Mann–Whitney *U* test)

Temperature recording started when the wasps could be permanently observed at the nests. As in our experiments *P. dominula* was observed early in spring (April) at the nests, relatively low temperatures (down to 0 °C) could be measured. *P. gallicus* was first observed in May at the nests and abandoned earlier in autumn. The frequency distribution of temperature categories (1 °C intervals) revealed microclimatic differences between the two locations (Fig. 6). As both data sets showed a similar skewedness in distribution, we calculated the median temperature for the time where measurements were conducted at both locations (14 May to 03 September 2013). It was significantly higher at the *P. gallicus* nests (Fig. 6 insert; *P. dominula*: 22.5 °C, *n* = 21,506; *P. gallicus*: 24.4 °C, *n* = 20,813; *P* < 0.0001, Mann–Whitney test).

**Fig. 7:**
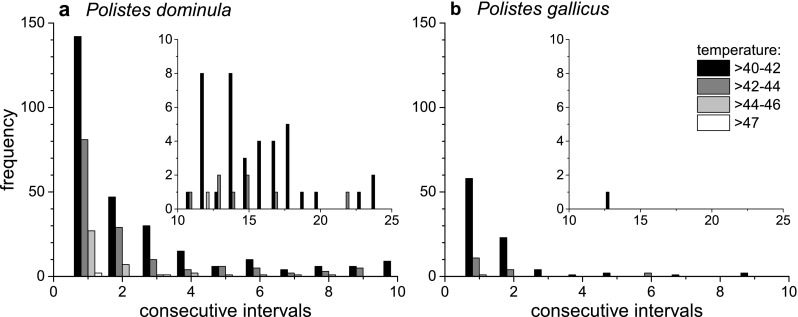
Frequency of consecutive intervals (15 min) of high temperatures (>42 to >47 °C) measured at two nests of *P. dominula* (**a**) and *P. gallicus* (**b**) during a breeding season in 2013 (14 May to 03 September). *Inserts* represent a frequency of consecutive intervals higher than 10. The nests of *P. dominula* were more frequently and longer exposed to higher temperatures than the nests of *P. gallicus*

The calculation of consecutive intervals (15 min) with a temperature above a certain threshold (42–47 °C) resulted mostly in a higher number for the *P. dominula* nest location in Austria (Fig. 7). In *P. dominula,* it could be observed once that in three consecutive intervals, 47 °C was exceeded. This high temperature is near their critical thermal maximum (activity CT_max_ = 47.1 °C, Table 1). The temperature at the nests of *P. gallicus*, which were outside in the recess of a window, never exceeded the threshold of 47 °C (activity CT_max_ = 47.6 °C).

## Discussion

In this study, we investigated local populations of the two closely related paper wasps, the Mediterranean *P. gallicus* in Tuscany, Italy, and *P. dominula* from a temperate region in Styria, Austria. We could demonstrate that the two species differ in thermal traits with regard to their environmental conditions. Specifically, they differed in their lower critical thermal limit (CT_min_ or onset of chill coma). In *P. gallicus*, onset of chill coma was at −0.4 °C, whereas in *P. dominula* this threshold was reached at -1.4 °C (Table 1). This is not surprising as we had expected a lower chill coma for the temperate than for the Mediterranean species. Observations and ambient temperature recordings at the nests (Fig. 5) revealed that individuals of *P. dominula* are to find at the nest early in spring and also late in autumn, when temperatures can drop below zero. The better tolerance of low temperatures is probably attributed to the higher climatic thermal variability in Central Europe and a necessary requirement for the successful distribution in harsher climate regions. Lancaster et al. (2015) could show in damselflies (*Ischnura elegans*) that the adaptive plasticity of lower thermal tolerances (i.e. acclimation ability) increased towards the northern latitude, expanding the range edge. A correspondence between the cold-hardiness of a species and the environmental thermal variation encountered has been found in several other comparative studies (see e.g. Gaston et al. 2009). Gibert et al. (2001) showed in temperate and tropical *Drosophila* species that chill-coma tolerance is a major climatic adaptation, and Andersen et al. (2015) demonstrated that chill-coma temperature and lower lethal temperature are the best predictors of cold distribution limits. However, chill-coma threshold is not a constant, but it is a dynamic thermal trait. The value of −1.4 °C determined for *P. dominula* in August was considerably higher than that of -3.0 °C determined in September by Käfer et al. (2015). Seasonality in cold resistance is an adaptation to thermal variability in temperate climate. For example, resistance of *Myrmica* ants to knock-down by cold and their rate of recovery after chill-coma was lower in summer than in autumn (Maysov and Kipyatkov 2011). Chill-coma recovery times in adult *Drosophila montana* flies showed a seasonal minimum between late autumn and early spring (Vesala et al. 2012). Seasonal variation of critical thermal limits could also be observed in Formosan and Eastern subterranean termites (Hu and Appel 2004).

Upper thermal limits vary less than lower limits among related species (see e.g. Gaston and Chown 1999; Kellermann et al. 2012; Hoffmann et al. 2012), and upper and lower thermal tolerance limits are often physiologically and evolutionarily decoupled, such that responses to cold stress rely on different physiological mechanisms than heat-stress responses (Chown et al. 2002), and may thus also evolve differently during range expansions. However, Gaston and Chown (1999) could show that the upper thermal tolerance (CT_max_) declines with an improvement in ability to tolerate low temperatures (i.e. a declining CT_min_) in dung beetles. These facts could be reasons why the results in the upper critical thermal maximum (CT_max_) revealed a trend, but no clear difference between the wasp species. The activity CT_max_ of *P. gallicus* was 0.5 °C higher than that of *P. dominula*, but the difference was not significant (Table 1, *P* = 0.24427, *t* test).

Microclimate temperature measurement at the nests should characterize environment conditions and reveal thermal adaptations. As expected, the median ambient temperature measured at the Mediterranean location in Italy was higher than that at the temperate location in Austria (Fig. 6, Italy 23.6 °C, Austria 18.8 °C). However, the highest ambient temperature (>47 °C) and the higher frequency when temperature exceeded 40 °C was not measured in Italy but in Austria (Fig. 6 and 7). This could be explained by the wasps’ differing nesting behaviour. In Austria, possibly due to lower minimal temperatures in spring and autumn, *P. dominula* mostly nests in well-sheltered habitats, for example under roof tiles inside lofts with connection to the outside. On hot summer days, the temperature can reach very high values at these locations. In contrast, *P. gallicus* avoids nesting at such closed sites. They will be found mostly outside, in sheltered places with nests oriented to the east, where they are exposed to the sun in the morning but not at the hottest time of day. As *P. dominula* originate from the Mediterranean climate region, it has developed physiological adaptations to high temperatures in evolutionary processes. *P. dominula* obviously did not lose its abilities when it dispersed to the north. However, recent extreme weather conditions due to climate warming elevated maximum temperatures in central Europe (APCC 2014), which challenge their thermoregulatory capacity. Analysis of environmental data at the nests (Fig. 5) revealed that 47 °C was exceeded in three consecutive intervals of 15 min (Fig. 7). This high temperature is well within the range of the wasps’ upper critical thermal limit (47.1 °C, Table 1), and therefore they have to invest time and energy for water foraging to cool their nest and larvae (see e.g. Steiner 1930; Kovac et al. 2009; Höcherl et al. 2016).

The climatic variability hypothesis postulates that species occurring in areas of low climatic variability have smaller thermal tolerance ranges than species living in a broad range of climatic conditions (Stevens et al. 2010). Sheldon and Tewksbury (2014) showed in different groups of dung beetles that thermal tolerance increases with seasonality and, therefore, climatic variability. Our results also confirm this hypothesis. The thermal tolerance range (CT_max_ − CT_min_) as a measure of the thermal fitness was 0.5 °C larger in *P. dominula* from Austria (higher climatic variability) than in *P. gallicus* from Italy (lower climatic variability). However, this difference seems to be too small to solely explain the successful dispersion of *P. dominula* in cooler climate regions. We suggest that other adaptations (to lower temperatures) in nesting and foraging behaviour could enable *P. dominula*’s successful dispersion. In addition, for the survival and distribution in temperate climates, the thermal tolerance of the overwintering queens should be a deciding and presumably the limiting factor for *P. gallicus*. This question remains to be investigated.

*Polistes dominula* wasps regulate the temperature of their nest actively by evaporative cooling and ‘passively’ by a careful site selection and the architecture of their nests (e.g. Steiner 1930; Höcherl et al. 2016). Our body temperature measurements at the nests delivered additional information on physiological and behavioural adaptations to microclimate conditions (Figs. 2 and 3). Individuals of *P. gallicus* exhibited and tolerated higher body temperatures than *P. dominula*. While *P. dominula* already tried to keep their thorax temperature below ambient air temperature at ~37 °C, in *P. gallicus* this threshold was not reached until ~41 °C (Fig. 3). Thus, *P. dominula* had to perform more cooling activity than *P. gallicus* to keep a lower body temperature. The cooling behaviour could explain why we could measure such high (lethal) ambient temperature at intact nests, which did not kill the individuals and destroy the colony. In *P. gallicus*, we suggest the ability to tolerate higher body temperatures to be an evolutionary adaptation to higher (mean) ambient temperature at their nesting sites (Fig. 6).

In our study, we could not draw conclusions from the species’ metabolism on climatic adaptations (Table 2; Fig. 4a). The species varied in metabolic response to temperature, but not in a consistent way. In the mass-specific standard or resting metabolism determined between 5 and 35 °C, differences could be detected in the slope of regressions (*P* < 0.05, ANOVA). Analysing investigated temperature categories revealed that at low (*T*_a_ = 5 °C) and high temperatures (*T*_a_ = 35 °C), *P. dominula* exhibited a higher metabolic performance, whereas at intermediate temperatures (*T*_a_ = 15 and 25 °C), the metabolism of *P. gallicus* was higher. This higher metabolism was probably caused by *P. gallicus’* higher thorax temperature (Table 2; Fig. 4b). At very high ambient temperatures (*T*_a_ = 40 and 45 °C), which were very stressful for the wasps, they were never at rest (just for a few seconds in the short 1-min measurement intervals) and exhibited a high energetic performance. The species did not differ in mass-specific metabolic rate at these extreme conditions. Though we are aware of the problem that resting and active metabolic rate are different physiological states, we also calculated regressions for the whole temperature range to allow the use of our data in models dealing with the impact of temperature on insect energetics. These functions may be seen as a representation of standard metabolic rate (SMR). Many insects do not show consistent rest at high temperature (our own observations). This does not mean, however, that they do not have an SMR.

In contrast to our results, Addo-Bediako et al. (2002) found that environmental temperature significantly influences interspecific variation in metabolic rate. A global-scale analysis of the standard metabolic rate of 346 insect species delivered evidence for a metabolic cold adaptation in insects. Insects from colder environments tend to have higher whole-organism metabolic rates. Vorhees et al. (2013) could confirm this result in a study on mosquitos (*Culex tarsalis*) from a cool-temperature, high-altitude site, which had significantly higher metabolic rates compared with two populations from warmer sites at each test temperature. However, in a similar study, by Terblanche et al. (2009), comparing the metabolic response to temperature of four populations of the tsetse fly *Glossina pallidipes*, collected across a range of climates in east Africa, only the population from the coolest site (elevation 1691 m) showed a metabolic curve slope significantly different from the other populations, and significant differences in metabolic rate (i.e. y-intercept) occurred only at the highest test temperature (32 °C). These results deliver evidence that there is no simple linear correlation between environmental (climatic) data and metabolic response. We suggest additional (e.g. behavioural) parameters to be responsible for the individuals’ metabolic performance.

In conclusion, we can say that there are differences in thermal tolerance traits in these two paper wasp species. However, the successful distribution of *P. dominula* could not only be explained by a significantly better thermal performance of its workers, but could be additionally caused by the queens’ thermal traits.
